# miR-17, miR-19b, miR-20a, and miR-106a are down-regulated in human aging

**DOI:** 10.1111/j.1474-9726.2010.00549.x

**Published:** 2010-04

**Authors:** Matthias Hackl, Stefan Brunner, Klaus Fortschegger, Carina Schreiner, Lucia Micutkova, Christoph Mück, Gerhard T Laschober, Günter Lepperdinger, Natalie Sampson, Peter Berger, Dietmar Herndler-Brandstetter, Matthias Wieser, Harald Kühnel, Alois Strasser, Mark Rinnerthaler, Michael Breitenbach, Michael Mildner, Leopold Eckhart, Erwin Tschachler, Andrea Trost, Johann W Bauer, Christine Papak, Zlatko Trajanoski, Marcel Scheideler, Regina Grillari-Voglauer, Beatrix Grubeck-Loebenstein, Pidder Jansen-Dürr, Johannes Grillari

**Affiliations:** 1 Aging and Immortalization Research, Department of Biotechnology, University of Natural Resources and Applied Life SciencesVienna, Austria, Muthgasse 18, A-1190 Vienna; 2 Departments of Immunology, Institute for Biomedical Aging Research, Austrian Academy of SciencesRennweg 10, 6020 Innsbruck, Austria (IBA); 3 Molecular and Cell Biology, Institute for Biomedical Aging Research, Austrian Academy of SciencesRennweg 10, 6020 Innsbruck, Austria (IBA); 4 Extracellular Matrix Research, Institute for Biomedical Aging Research, Austrian Academy of SciencesRennweg 10, 6020 Innsbruck, Austria (IBA); 5 Endocrinology, Institute for Biomedical Aging Research, Austrian Academy of SciencesRennweg 10, 6020 Innsbruck, Austria (IBA); 6 Department of Natural Sciences, Institute of Physiology, University of Veterinary Medicine ViennaVeterinärplatz 1, A-1210 Wien, Austria; 7 Department of Genetics, University of SalzburgHeilbrunnerstraße 34, 5020 Salzburg, Austria; 8 Department of Dermatology, Medical University of ViennaA-1090 Vienna, Austria; 9 Department of Dermatology, SALK and Paracelsus Medical UniversitySalzburg, Austria; 10 Institute for Genomics and Bioinformatics and Christian Doppler Laboratory for Genomics and Bioinformatics, Graz University of TechnologyPetersgasse 14, 8010 Graz, Austria

**Keywords:** aging, miR-106a, miR-17, miR-17-92 cluster, miR-19b, miR-20a, miRNA microarray, p21 (CDKN1A), senescence

## Abstract

Aging is a multifactorial process where deterioration of body functions is driven by stochastic damage while counteracted by distinct genetically encoded repair systems. To better understand the genetic component of aging, many studies have addressed the gene and protein expression profiles of various aging model systems engaging different organisms from yeast to human. The recently identified small non-coding miRNAs are potent post-transcriptional regulators that can modify the expression of up to several hundred target genes per single miRNA, similar to transcription factors. Increasing evidence shows that miRNAs contribute to the regulation of most if not all important physiological processes, including aging. However, so far the contribution of miRNAs to age-related and senescence-related changes in gene expression remains elusive. To address this question, we have selected four replicative cell aging models including endothelial cells, replicated CD8^+^ T cells, renal proximal tubular epithelial cells, and skin fibroblasts. Further included were three organismal aging models including foreskin, mesenchymal stem cells, and CD8^+^ T cell populations from old and young donors. Using locked nucleic acid-based miRNA microarrays, we identified four commonly regulated miRNAs, miR-17 down-regulated in all seven; miR-19b and miR-20a, down-regulated in six models; and miR-106a down-regulated in five models. Decrease in these miRNAs correlated with increased transcript levels of some established target genes, especially the cdk inhibitor p21/CDKN1A. These results establish miRNAs as novel markers of cell aging in humans.

Continuous stochastic damage contributes to the gradual attenuation of physiologic functions, which are partially alleviated by genetically encoded repair systems (Kirkwood, 2008). Genetic determinants, which have a direct impact on these biological processes can be characterized by either studying model organisms that are amenable to genetic manipulations (Lepperdinger *et al.*, 2008), or by applying functional genomic methods. Both strategies have helped to identify genes and proteins, which potentially modulate the aging process. Proteins identified in various studies have been compiled in a database only recently (de Magalhaes *et al.*, 2009). Yet hardly any study has so far addressed the role of miRNAs during aging (Bates *et al.*, 2009; Grillari & Grillari-Voglauer, 2010).

MiRNAs are a class of small non-coding silencing RNAs of approximately 22 nucleotides in length (Ghildiyal & Zamore, 2009). They confer specificity to the RNA-induced silencing complex that either degrades or translationally represses target mRNAs (Bhattacharyya *et al.*, 2006; Carthew & Sontheimer, 2009). As the recognition of target mRNAs mainly depends on the small seed region within the mature miRNA, a single miRNA potentially regulates up to several hundred mRNA targets, thus orchestrating a large variety of cellular processes (Lim *et al.*, 2005; Stefani & Slack, 2008).

Here, we have set out to systematically compare miRNA transcription profiles in old vs. young human cells. Employed were *in vitro* replicative senescence of endothelial cells (Chang *et al.*, 2005; Hampel *et al.*, 2006), renal proximal tubular epithelial cells (Wieser *et al.*, 2008), skin fibroblasts (Hutter *et al.*, 2002; Stockl *et al.*, 2006) as well as an intra-individual comparison of *in vivo* replicatively exhausted CD8^+^ T cells (Saurwein-Teissl *et al.*, 2002; Effros *et al.*, 2005). Furthermore, we used bone-derived mesenchymal stem cells (Fehrer *et al.*, 2007; Laschober *et al.*, 2009), foreskin (Oender *et al.*, 2008), as well as CD8^+^ CD28^+^ T cells from old versus young donors (Lazuardi *et al.*, 2009). Detailed characterization of these models as well as an overview over biological and technical replicates and experimental design is presented in the supplements (Figs S1 and S2a). Experiments were approved by the local ethical committees, and written informed consent is available from all donors.

Locked nucleic acid (LNA)-miRNA microarrays were spotted (Castoldi *et al.*, 2006) using Sanger miRBase v9.2 (Griffiths-Jones *et al.*, 2008) probe sets consisting of 559 human, 170 mouse, and 77 not yet annotated (miRPlus sequences; Exiqon Inc., Vedbaek, Denmark) miRNA probes. Microarray design, a comprehensive set of related protocols as well as raw and normalized intensity data have been submitted to Array Express Database compliant to Minimum Information About a Microarray Experiment standards (Brazma *et al.*, 2001; Brazma, 2009). For Array Express accession numbers and detailed materials and methods see Data S1 and Fig. S2a.

Depending on the experimental system, statistical analysis identified 10–20% of the miRNAs as regulated, while the majority remained unchanged during aging (Table S1). Applying hierarchical clustering of all regulated miRNAs, members of the miR-17-92 cluster and paralogous clusters stood out as being commonly down-regulated (Fig. S3).

Using linear models and moderated T-statistics (Smyth, 2004) in combination with false discovery rate (FDR) adjustment according to Hochberg-Benjamini (1990), we calculated differential expression of miRNAs (FDR-adjusted *P*-value < 0.05) for each of the seven model systems independently (available as tab-delimited sheet in Table S2). Intersections of these lists of regulated miRNAs were then analyzed for the replicative aging models and the *ex vivo* models, which resulted in five miRNAs with common expression changes in replicative aging, and 34 miRNAs that changed expression during organismal aging (Fig. 1a). Interestingly, both replicative and organismal aging share a common set of four miRNAs (‘public miRNAs’) that belong to the miR-17-92 cluster or its paralogous cluster miR-106a-363 and are down-regulated in both conditions (Fig. 1b). Figure 1b shows that microarray analysis identified miR-17 as significantly down-regulated in all seven model systems, while miR-19b and miR-20a are down-regulated in six and miR-106a in five of the seven model systems. Interestingly, three of these four miRNAs also share the same seed sequence (Fig. 1c) indicating a cooperative relief of translational inhibition of a common and important set of target genes. Although low transcription levels of mature miR-17 have been found in S phase cells compared to G0/G1 and G2/M phase HeLa cells (Cloonan *et al.*, 2008), our result is not a mere growth arrest phenomenon, as miR-17 and miR-19b are not regulated in young replicating vs. quiescent endothelial cells (data not shown).

**Fig. 1:**
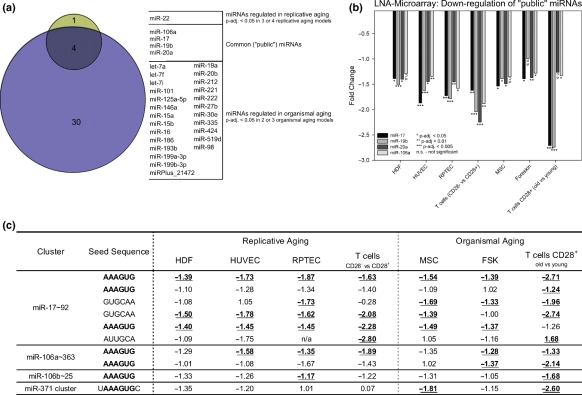
Microarray analysis of differential expression and enrichment of regulated miRNAs in replicative and organismal aging. (a) Size-adjusted Venn diagram depicting the intersection of regulated miRNAs from replicative and organismal aging models: the upper, yellow circle represents five miRNAs that were found significantly regulated (false discovery rate-adjusted *P*-value < 0.05) in at least three of the four replicative models. Only miRNAs with uniform up- or down-regulation in all models were considered. For organismal aging experiments, 34 miRNAs were significantly regulated (FDR-adjusted *P*-value < 0.05) in at least two of three models, indicated by the lower, purple circle. (b) The intersection contains three miRNAs of the miR-17-92 cluster, namely miR-17, miR-19b, and miR-20a, as well as miR-106a of the paralogous miR-106a-363 cluster. The individual ‘old vs. young’ ratios for these miRNAs, calculated from microarray data, are depicted in a barchart. (c) Fold changes in transcription of young vs. old based on microarrays are given for all members of the miR-17-92 cluster as well as selected miRNAs from paralogous clusters together with 5′ seed sequences. Adjusted *P*-values < 0.05 are marked in bold and underlined format, indicating statistically significant regulation.

Using quantitative polymerase chain reaction, we confirmed the down-regulation of miR-17, miR-19b, miR-20a, and miR-106a (Fig. 2a) but observed a greater dynamic range in fold changes ranging up to a 6-fold down-regulation, thus indicating an even stronger decrease in the transcription of all four miRNAs with age.

**Fig. 2:**
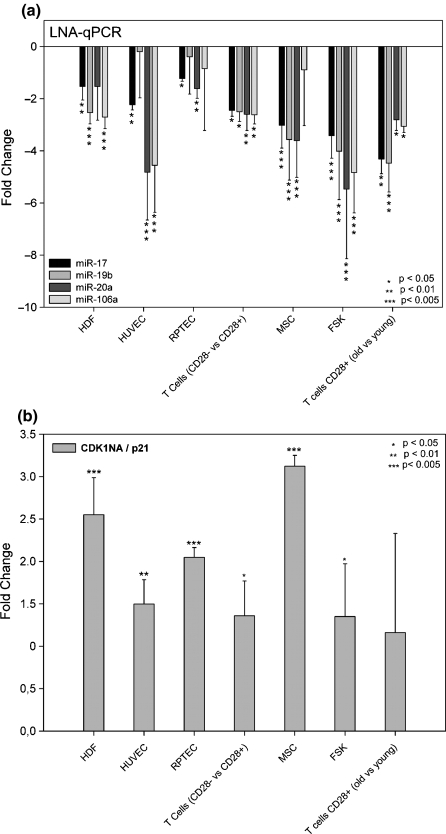
Quantitative real-time polymerase chain reaction (PCR) of miRNAs and the published target p21/CDKN1A. (a) Down-regulation of miR-17, miR-19b, miR-20a, and miR-106a in microarray experiments was validated by quantitative PCR analysis. miRNA expression values were normalized to GAPDH levels for each experiment (*n* = 8; *P* < 0.05, one sample *t*-test with μ_0_ = 0) (b) Messenger RNA levels of p21/CDKN1A were analyzed by quantitative polymerase chain reaction and normalized to GAPDH expression levels (*n* = 8; *P* < 0.05, one sample *t*-test with μ_0_ = 0). Increased p21 levels were observed in senescence and organismal aging indicating negative correlation to transcription of members of the miR-17-92 cluster. HDF: human diploid fibroblasts, HUVEC: human umbilical vein endothelial cells, RPTEC: renal proximal tubular epithelial cells, MSC: bone marrow derived mesenchymal stem cells, FSK: human foreskin, GAPDH: Glycerinaldehyd-3-phosphat-Dehydrogenase.

Down-regulation of members of the miR-17-92 cluster has been reported recently in age-related conditions like in stress-induced senescence (Li *et al.*, 2009a), after p53 induction (Brosh *et al.*, 2008) as well as after low level irradiation (Maes *et al.*, 2008a,b;) of human fibroblasts.

However, other reports on differential expression of miRNAs in replicative senescence of fibroblasts (Brosh *et al.*, 2008; Lal *et al.*, 2008; Bhaumik *et al.*, 2009; Maes *et al.*, 2009), in senescence of mesenchymal stem cells (Wagner *et al.*, 2008), of human and mouse brain tissue (Lukiw, 2007; Li *et al.*, 2009b) as well as of murine liver (Maes *et al.*, 2008a,b;) and lung (Williams *et al.*, 2007; Izzotti *et al.*, 2009) do not explicitly report regulation of miR-17-92 miRNAs. This might either be because of the fact that in some studies only up-regulated miRNAs have been reported or to the strategy that only at least 2-fold changed miRNAs were considered.

As the ability of array platforms to detect fold changes down to 1.3-fold (Wurmbach *et al.*, 2003) and because LNA-based microarray methodology is considered to be one of the most sensitive and reliable at the moment (Willenbrock *et al.*, 2009), we included miR-17-92 cluster members that are significantly regulated even at levels below 2-fold, even more so, because array results also are reported to have the tendency to underestimate the ratios (Wurmbach *et al.*, 2003).

Interestingly, among the published targets of the miR-17-92, cluster is p21/CDKN1A mRNA (Ivanovska *et al.*, 2008; Inomata *et al.*, 2009). Indeed, p21/CDKN1A mRNA levels are negatively correlated in all model systems (Fig. 2b), although the ratio in old vs. young T cells does not reach significance.

These data indicate that the miR-17-92 cluster, which is known to contribute to transcriptional regulation in cell cycle control and tumorigenesis (He *et al.*, 2005), also contributes to transcriptional regulation in senescence and aging, consistent with the known interdependence between senescence and tumorigenesis (Campisi, 2003; Rodier *et al.*, 2007).

Among others, E2F transcriptionally activates (Woods *et al.*, 2007) and p53 represses the miR-17-92 cluster (Yan *et al.*, 2009). Thus, decreased miR-17-92 levels are consistent with the notion that, E2F family members decrease (Dimri *et al.*, 1994), while p53 activity increases in senescence (Atadja *et al.*, 1995; Kulju & Lehman, 1995).

Downstream of miR-17-92 are 19 experimentally confirmed mRNA targets besides p21. Many of them are involved in tumorigenesis and cell cycle control (Table S3). Indeed, miR-17-92 suppression induces growth arrest in anaplastic thyroid cancer cell models (Takakura *et al.*, 2008). Furthermore, an increase in the level of miR-17-92 is associated with a decrease in ROS and DNA damage in RB mutated tumor cells (Ebi *et al.*, 2009). It will be exciting to test in an experimental system whether a decrease in miR-17-92, as detected in our models of aging, conversely results in more ROS and DNA damage, both well accepted driving forces of age-related functional decline.

In summary, our results implicate specific changes of miRNA abundance and activity in a broad range of human aging models and suggest the use of miR-17, miR-19b, miR-20a, and miR-106 as novel biomarkers of cellular aging.

## Supporting Information

**Fig. S1** Characteristization of the analyzed model systems of aging.

**Fig. S2** Experimental design of differential miRNA analysis.

**Fig. S3** Heatmap visualization and clustering of miRNA expression data.

**Table S1** Overview on total numbers of transcribed miRNA as well on miRNAs detected as differentially transcribed

**Table S2** Compilation of all regulated miRNAs in all experimental systems (provided as MS Excel file only).

**Table S3** Experimentally validated target mRNAs of the miR-17-92 cluster.

**Data S1** Supporting materials and methods.

As a service to our authors and readers, this journal provides supporting information supplied by the authors. Such materials are peer-reviewed and may be re-organized for online delivery, but are not copy-edited or typeset. Technical support issues arising from supporting information (other than missing files) should be addressed to the authors.
