# Supplementary figures and images for: miR-17, miR-19b, miR-20a, and miR-106a are down-regulated in human aging
Source: Aging Cell. 2010 Apr;9(2):291–6. doi: 10.1111/j.1474-9726.2010.00549.x (PMC2848978; doi:10.1111/j.1474-9726.2010.00549.x)

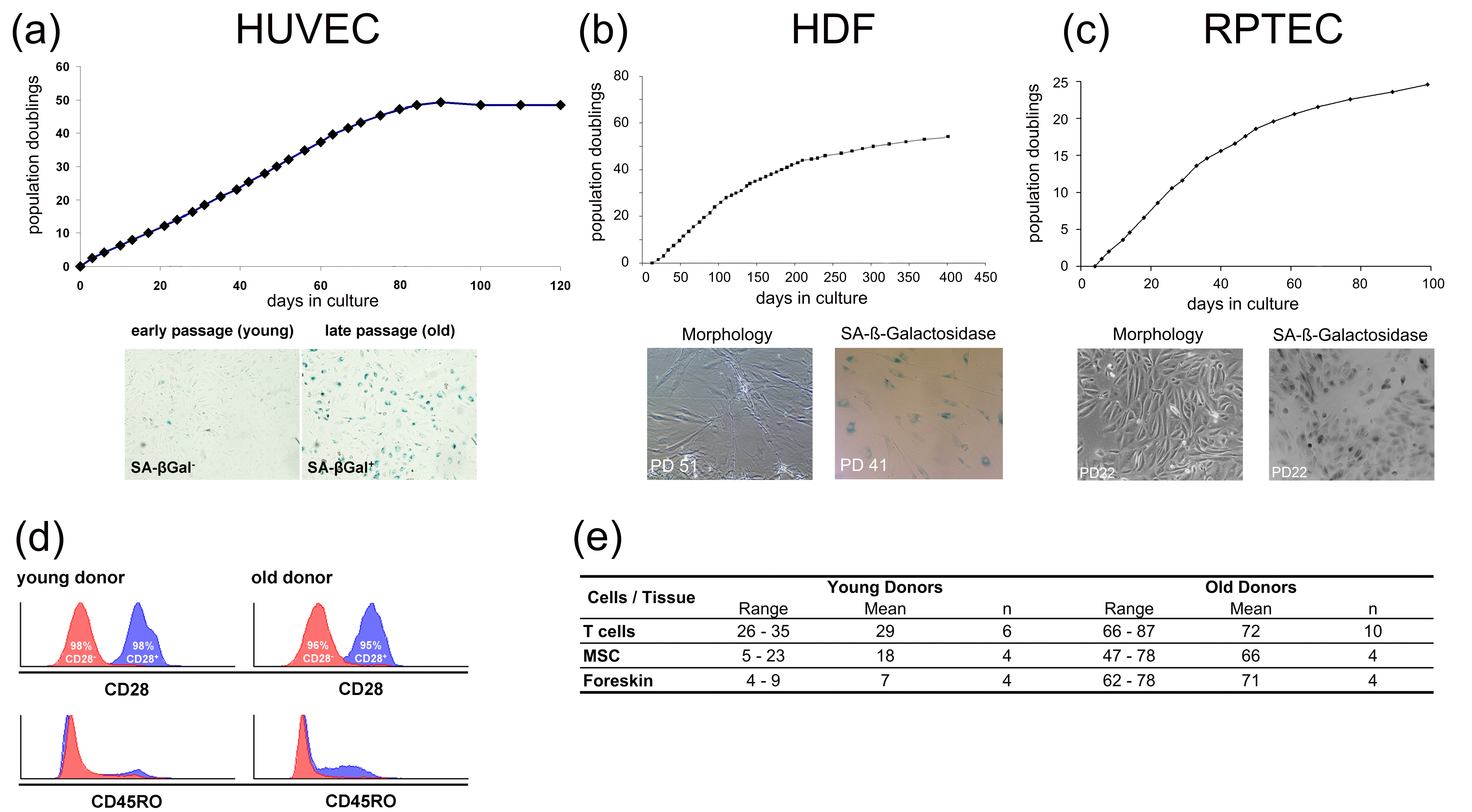

Supplement: Supplementary file 1 [file ace0009-0291-SD1.tif]

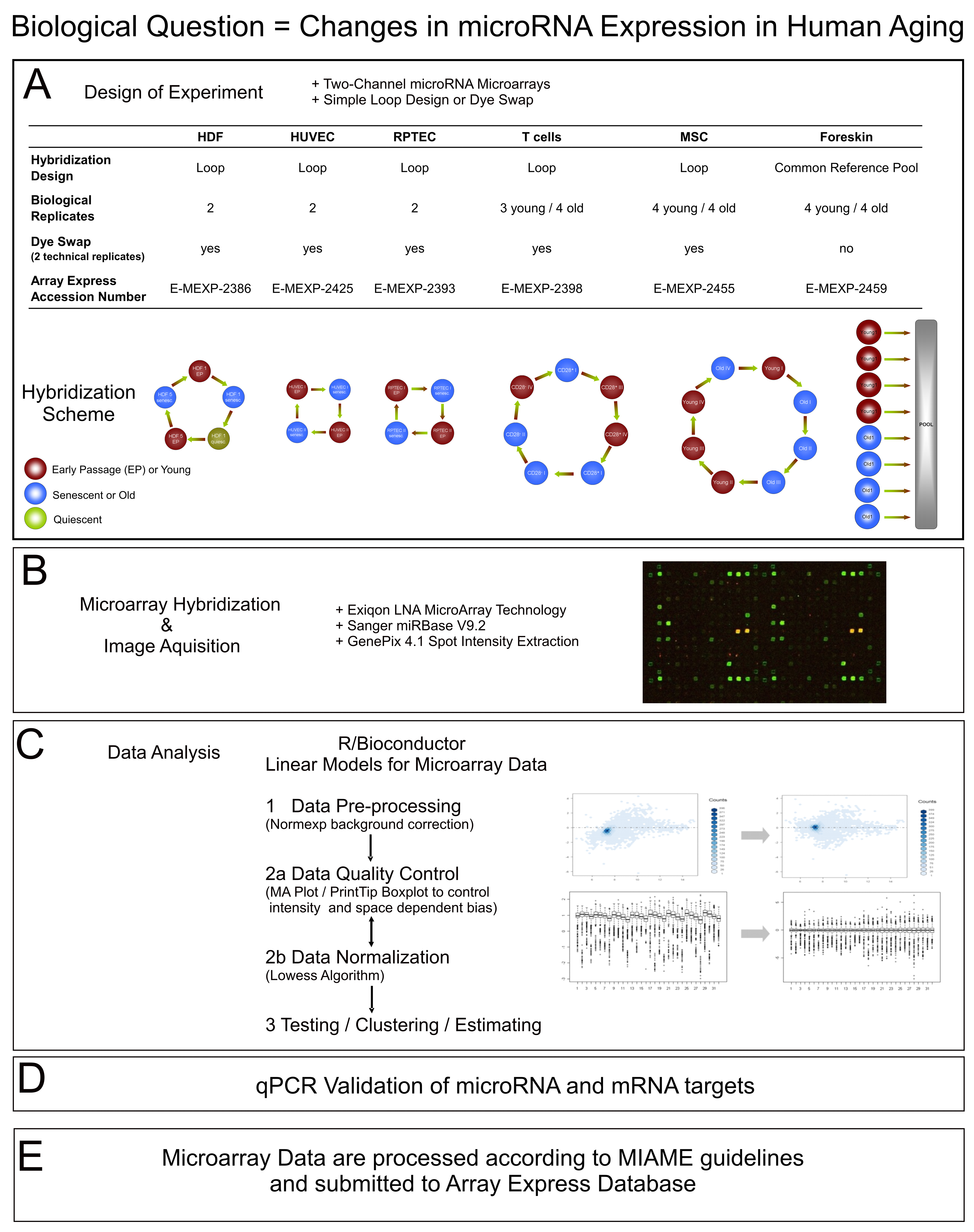

Supplement: Supplementary file 2 [file ace0009-0291-SD2.tif]

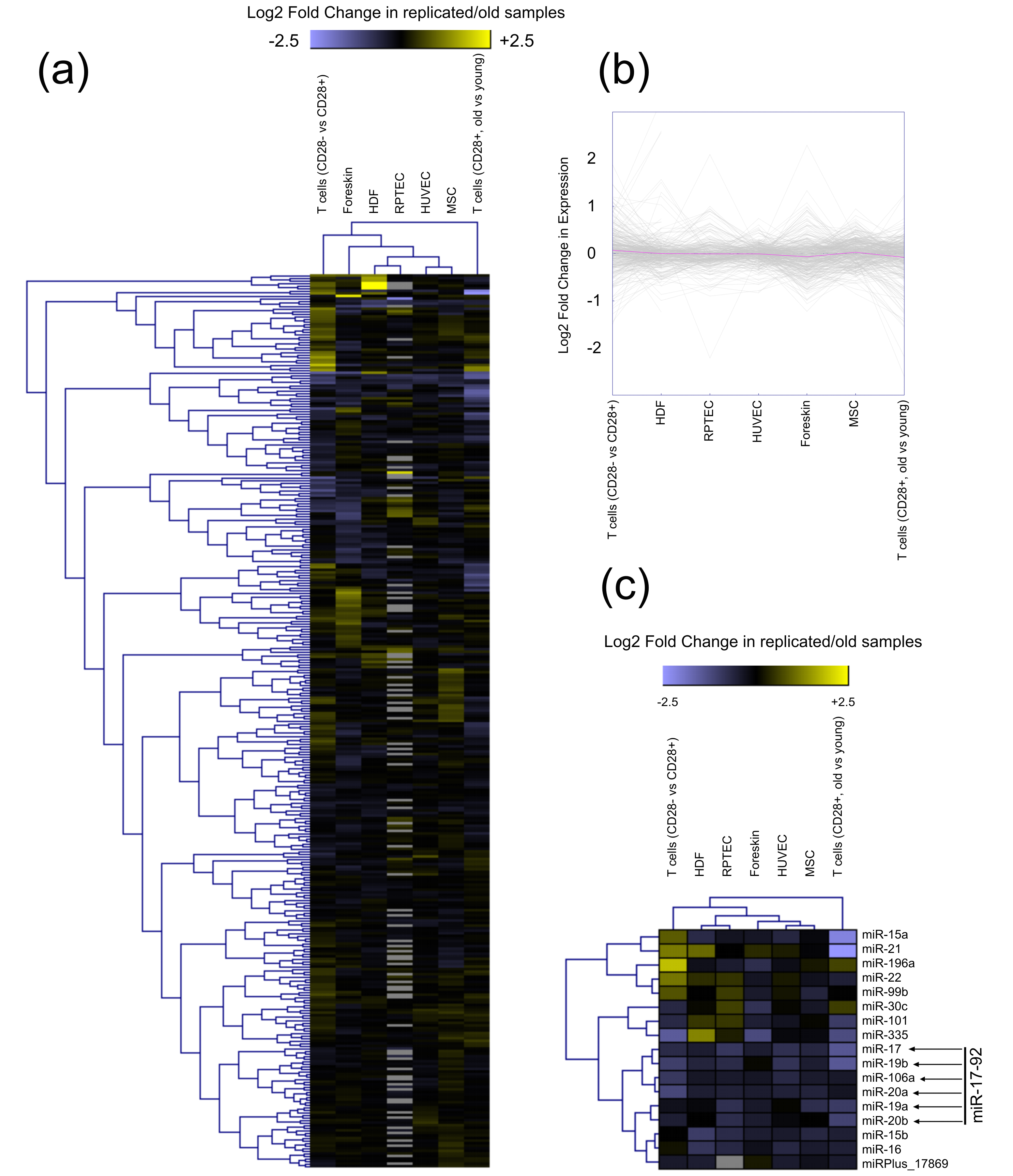

Supplement: Supplementary file 3 [file ace0009-0291-SD3.tif]

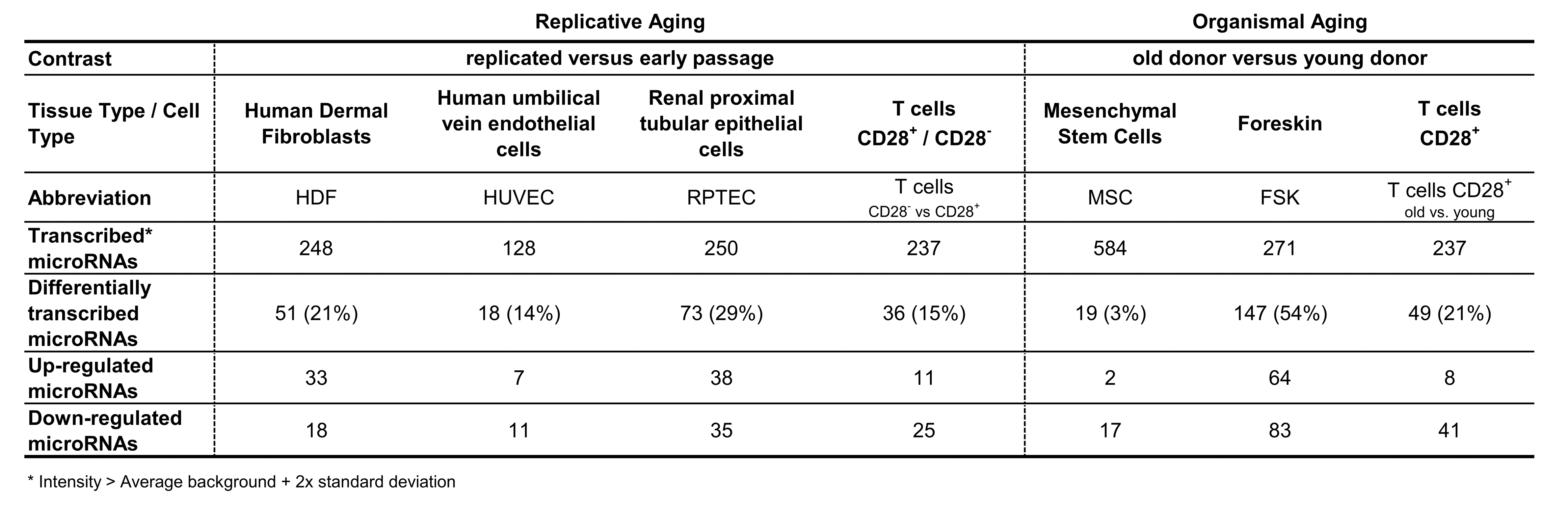

Supplement: Supplementary file 4 [file ace0009-0291-SD4.tif]

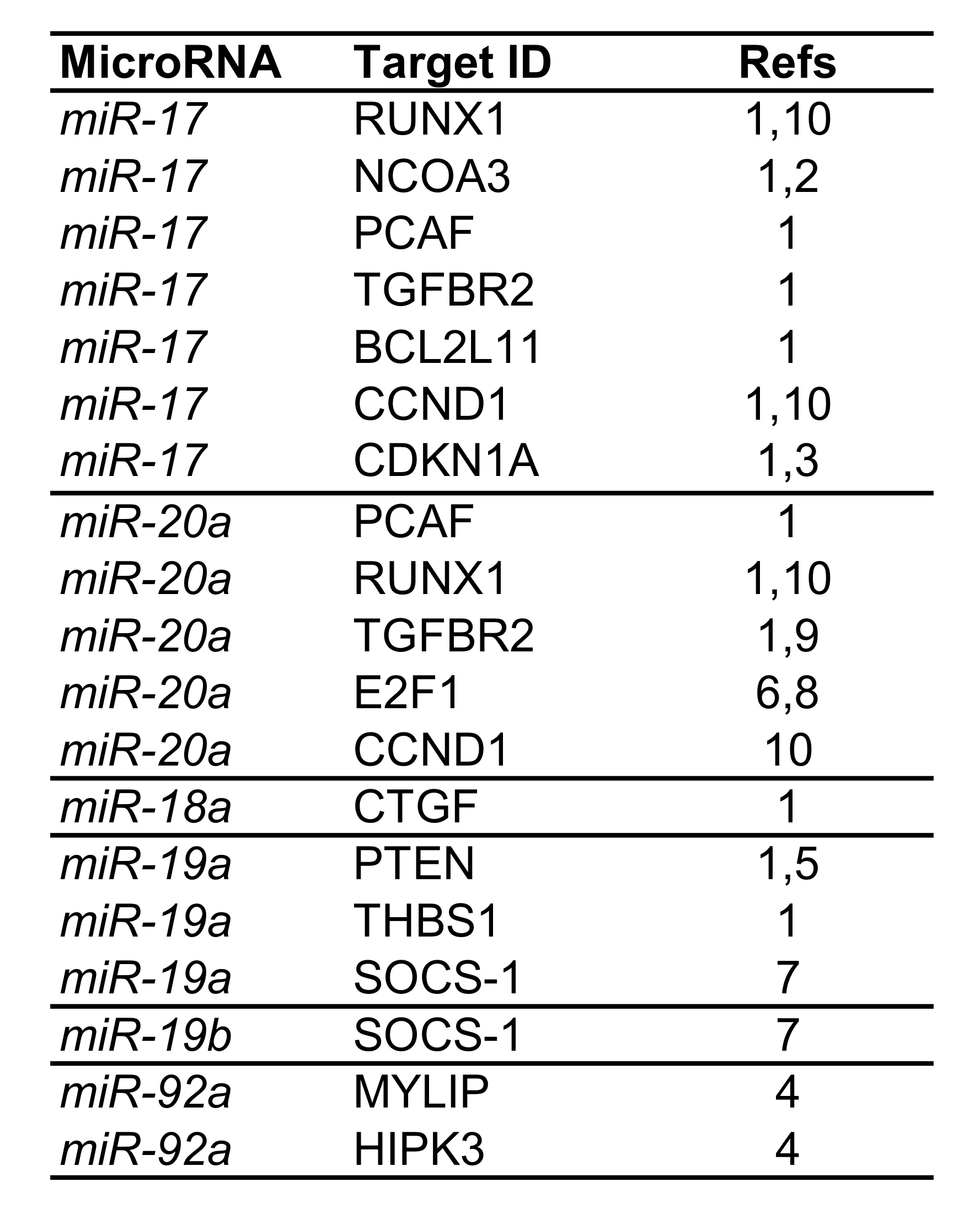

Supplement: Supplementary file 5 [file ace0009-0291-SD5.tif]
