# Supplementary material for: miR-17, miR-19b, miR-20a, and miR-106a are down-regulated in human aging
Source: Aging Cell. 2010 Apr;9(2):291–6. doi: 10.1111/j.1474-9726.2010.00549.x (PMC2848978; doi:10.1111/j.1474-9726.2010.00549.x)
Supplement: Supplementary file 7 [file ace0009-0291-SD7.doc]

**Figure S1: Characteristization of the analyzed model systems of aging.** Representative growth curves of HUVECs (a), fibroblasts (b), and RPTECs (c) are presented, indicating replicative growth arrests at late passages. Senescence at the time of harvest was confirmed by >95% positive cells after senescence-associated β-galactosidase staining. Furthermore, morphological characteristics were typical for senescent cells. Representative images of cells are given below the growth curves. (d) Replicatively exhausted (CD28^-^) versus non-exhausted CD8^+^ T cells (CD28^+^) were separated after taking blood samples by magnetic-activated cell separation and identity and purity of the populations was confirmed by flow cytometry using antibodies against CD28. (e) Donor characteristics regarding the age range, the mean age for young and old donors as well as the total number (n) of donors for T cells, mesenchymal stem cell, and foreskin specimens are summarized.

Knapen D, Vergauwen L, Laukens K, Blust R (2009) Best practices for hybridization design in two-colour microarray analysis. *Trends Biotechnol* **27,** 406-414.

**Figure S2: Experimental design of differential miRNA analysis.** (a) Depending on the individual experimental setup (the number of biological replicates and the contrasts that were of interest) an appropriate hybridization strategy was chosen for each aging model system. Experimental designs of all hybridizations are depicted using a visualization scheme introduced by (Knap*en et a*l., 2009). In addition to the number of biological and dye swap replicates, also the Array Express accession numbers are given, which allow access to the entire set of raw and normalized microarray data. (b) According to the chosen design, total RNA was hybridized to Sanger miRBase v8.0 or v9.2 LNA microarrays and scanned as described in the supporting material and methods section. (c) For data analysis array spot intensities were first log_2_ transformed, background corrected using the normexp algorithm and lowess normalized using R in combination with LIMMA (Linear Models for Microarray Data, Bioconductor). A representative graph before and after normalization is shown. Normalized data were then visualized and clustered using Genesis software from TU Graz, and tested for differential expression by fitting linear models to the data and applying moderated t-statistics and false discovery rate adjustment. (d) Data regarding differential expression of miRNAs were confirmed using LNA qPCR. In addition, expression of selected mRNA targets of the regulated miRNAs was measured using SYBR Green qPCR. (e) Comprehensive protocols and data for all miRNA microarray experiments were submitted to Array Express (http://www.ebi.ac.uk/microarray-as/ae/) according to the Minimal Information about Microarray Data (MIAME) submission guidelines.

**Figure S3: Heatmap visualization and clustering of miRNA expression data.** (a) The fold changes in expression (old versus young) of the miRNAs were log_2_-transformed and visualized in a heatmap where yellow color indicates up-regulation in aged or replicated samples, while blue corresponds to down-regulation (maximum yellow/blue color intensities were manually set to three). Experiments as well as miRNAs were subjected to hierarchical clustering (complete linkage distance calculation) indicated by dendrograms. (b) Linked expression view of log_2_ transformed fold changes in miRNA expression (old vs. young or replicated vs young) is shown. The pink line, corresponding to the median expression change in each model, indicates that the majority of miRNAs exhibits no regulation (log_2_ fold change = 0). The entire set of miRNAs was filtered for miRNAs with altered transcription (+/- 0.4 log_2_-fold change) in the majority of the single model systems resulting in 17 miRNAs. (c) A heatmap of these 17 miRNAs after hierarchical clustering is shown. Clustering yielded a 9 miRNAs comprising cluster characterized by consistent down-regulation in the majority of experiments. miRNAs generated from a single primary transcript at 13q31.3 – the miR-17-92 cluster of miRNAs – (or paralogous clusters 106a-363 and 106b-25) were found to be enriched among these miRNAs and are highlighted by black arrows.

**Table S1: Overview on total numbers of transcribed miRNA as well on miRNAs detected as differentially transcribed.** From a total set of 806 miRNAs, those with transcription levels 2-fold above background were considered present and subjected to statistical testing. miRNAs were considered to be differentially transcribed if a false discovery rate adjusted p-value of below 0.05 was found.

HUVEC: human umbilical vein endothelial cells; HDF: normal human diploid fibroblasts; RPTEC: renal proximal tubular epithelial cells; MSC: bone marrow derived mesenchymal stem cells; FSK: human foreskin; RA: replicative aging; OA: organismal aging

**Table S2: Compilation of all regulated miRNAs in all experimental systems (provided as MS Excel file only).** Each model system is presented in a separate excel sheet.

**Table S3: Experimentally validated target mRNAs of the miR-17-92 cluster.** References within the table correspond to following within the main text:

(1) Cloonan N, Brown MK, Steptoe AL, Wani S, Chan WL, Forrest AR, Kolle G, Gabrielli B, Grimmond SM (2008) The miR-17-5p microRNA is a key regulator of the G1/S phase cell cycle transition. *Genome Biol* **9,** R127.

(2) Hossain A, Kuo MT, Saunders GF (2006) Mir-17-5p regulates breast cancer cell proliferation by inhibiting translation of AIB1 mRNA. *Mol Cell Biol* **26,** 8191-8201.

(3) Ivanovska I, Ball AS, Diaz RL, Magnus JF, Kibukawa M, Schelter JM, Kobayashi SV, Lim L, Burchard J, Jackson AL*, et al.* (2008) MicroRNAs in the miR-106b family regulate p21/CDKN1A and promote cell cycle progression. *Mol Cell Biol* **28,** 2167-2174.

(4) Landais S, Landry S, Legault P, Rassart E (2007) Oncogenic potential of the miR-106-363 cluster and its implication in human T-cell leukemia. *Cancer Res* **67,** 5699-5707.

(5) Lewis BP, Shih IH, Jones-Rhoades MW, Bartel DP, Burge CB (2003) Prediction of mammalian microRNA targets. *Cell* **115,** 787-798.

(6) O'Donnell KA, Wentzel EA, Zeller KI, Dang CV, Mendell JT (2005) c-Myc-regulated microRNAs modulate E2F1 expression. *Nature* **435,** 839-843.

(7) Pichiorri F, Suh SS, Ladetto M, Kuehl M, Palumbo T, Drandi D, Taccioli C, Zanesi N, Alder H, Hagan JP*, et al.* (2008) MicroRNAs regulate critical genes associated with multiple myeloma pathogenesis. *Proc Natl Acad Sci U S A* **105,** 12885-12890.

(8) Pickering MT, Stadler BM, Kowalik TF (2009) miR-17 and miR-20a temper an E2F1-induced G1 checkpoint to regulate cell cycle progression. *Oncogene* **28,** 140-145.

(9) Volinia S, Calin GA, Liu CG, Ambs S, Cimmino A, Petrocca F, Visone R, Iorio M, Roldo C, Ferracin M*, et al.* (2006) A microRNA expression signature of human solid tumors defines cancer gene targets. *Proc Natl Acad Sci U S A* **103,** 2257-2261.

(10) Yu Z, Wang C, Wang M, Li Z, Casimiro MC, Liu M, Wu K, Whittle J, Ju X, Hyslop T*, et al.* (2008) A cyclin D1/microRNA 17/20 regulatory feedback loop in control of breast cancer cell proliferation. *J Cell Biol* **182,** 509-517.

(11) Li G, Luna C, Qiu J, Epstein DL, Gonzalez P (2009) Alterations in microRNA expression in stress-induced cellular senescence. *Mech Ageing Dev*.

**Overview on Supporting Information**

Table S1

Table S2

Table S3

Fig. S1

Fig. S2

Fig. S3

Legends are included in the fig. legends section above.

Supporting materials and methods

**Supporting Material and Methods**

**Cell Lines**

**Human Dermal Fibroblasts (HDFs)**

Normal human fibroblasts were isolated from skin biopsies of two different healthy causcasian patients, a 41-years old female (HDF-1) and a 16-years old male (HDF-5). HDF-5 and HDF-1 cells were cultivated in DMEM/HAM’s F-12 medium (Biochrom KG, Berlin, Germany) supplemented with 10% fetal calf serum (FCS) 4 mM L-glutamine (Sigma) and Primocin antibiotics (100 µg/mL). Cells were grown at 37 °C in ambient atmosphere containing 5% CO_2_. Until confluence was reached HDF cells were routinely passaged at 1:4 or 1:3 split ratios. Upon decrease in growth rate and entry of irreversible growth arrest split ratio was changed to 1:2 and after cells had stopped dividing, fresh medium was added every 7 days. HDF-1 cells reached senescence after 54 population doublings (PDLs), while HDF-5 cells had stopped growing after 72.7 PDLs.

**Human Umbilical Vein Endothelial Cells (HUVECs)**

Endothelial cells were isolated from human umbilical veins (Jaf*fe et a*l., 1973) and cultured in Endothelial Cell Basal Medium (Lonza) supplemented with EGM Single Quots (Lonza), containing hEGF 0.5 mL, hydrocortisone 0.5 mL, GA-1000 0.5 mL, BBE 2.0 mL, FBS 10.0 mL. The cells were subcultured by trypsinization with trypsin-EDTA (Gibco Life Technologies, Vienna, Austria), seeded on cell culture dishes coated with 0.2% gelatine and grown at 37 °C at ambient atmosphere containing 5% CO_2_. Cells were passaged at a ratio of 1:5 in regular intervals. At later passages, the splitting ratio was reduced to 1:3 and 1:2, respectively. Cells were passaged before reaching 70-80% confluency. PDL were estimated using the following equation: PDL = (log_10_(F)−log_10_(I)) / 0.301 (where F is the number of cells at the end of one passage, and I the number of cells that were seeded at the beginning of one passage). After roughly 50 population doublings, cells had reached growth arrest.

**Renal Proximal Tubular Epithelial Cells (RPTECs)**

RPTECs were cultivated as recently reported (Wies*er et a*l., 2008). In brief, within 24 hours after surgery tissue from the renal cortex was fragmented and incubated at 37°C for 15-20 minutes in DMEM/Ham’s F12 (1:1) (Biochrom KG, Berlin, Germany) containing 1 mg/mL collagenase type IV (PAN-BioTech GmbH, Aidenbach, Germany) and 1 mg/mL trypsin-inhibitor (Sigma, Vienna, Austria). After being passed through a 105 µm nylon mesh the filtrate was centrifuged, washed twice with phosphate buffered saline (PBS), resuspended in medium and dispensed into roux-flasks (Nunc, Wiesbaden, Germany). 24 hours thereafter medium was changed. The initial passage of confluent cells after 3-5 days was considered as PDL zero. Cells were passaged (1:2 to 1:4) at confluence, using 0.25% trypsin/0.02% EDTA, which was inactivated with 1 mg/mL trypsin-inhibitor. Cumulative PDL was calculated as a function of passage number and split ratio. Medium consisted of DMEM/Ham’s F12 (1:1) supplemented with 4 mM L-glutamine, 10 mM HEPES buffer, 5 pM triiodothyronine, 10 ng/mL recombinant human EGF, 3.5 µg/mL ascorbic acid, 5 µg/mL transferrin, 5 µg/mL insulin, 25 ng/mL prostaglandin E1, 25 ng/mL hydrocortisone and 8.65 ng/mL sodium selenit (all from Sigma). For RPTEC/TERT1 the medium was supplemented with 100 µg/mL G418 (Sigma).

**In vivo specimens**

**Mesenchymal Stem Cells**

MSC were isolated from the iliac crest of systemically healthy individuals (young donors, n=4, mean age 18, range 5-23; elderly donors, n=4, mean age 66, range 47-78) which had been harvested for reconstructive bone surgery of defects within other areas of the body as described previously (Fehr*er et a*l., 2007). Briefly, a small biopsy of substantia spongiosa osseum, which otherwise would have been discarded based on necessary bone for molding and recontouring prior to insertion into the recipient site was taken to further investigation under an Institutional Review Board-approved protocol after having obtained patients’ written consent. After surgery, the bone was transferred into minimal essential medium (MEM) supplemented with 20% heat-inactivated fetal calf serum, 100 units/mL penicillin, 100 μg/mL streptomycin (growth medium) for transportation from the operation theatre to the clean room at room temperature. The biopsies were fragmented and marrow cells were isolated from pieces (20-100 mm^3^) by centrifugation (400*xg*, 1 minute). After centrifugation, the remaining pieces were treated with collagenase (2.5 mg/mL in MEM) for 2-3 hours at 37°C, 20% O_2_ and 5% CO_2_. Thereafter, the specimen was again centrifuged (400*xg*, 1 minute). Cells were resuspended and loaded on a Ficoll-Paque Plus® gradient and centrifuged at 2,500*xg* for 30 minutes. Cells were harvested from the interphase (density <1.075 g/mL), washed and collected by centrifugation (1,500*xg*, 15 minutes). Cells were cultured at a density of 0.2 – 0.5 x 10^6^ cells/cm^2^ at 5% CO_2_ and 37°C and 3% O_2_ (Thermo Electron Forma Series II, 3110). After 24 hours, the non-adherent cell fraction was removed by washing twice with phosphate-buffered saline (PBS). After the primary culture had reached approximately 30 – 50% confluence, cells were washed twice with PBS, and subsequently treated with 0.05% trypsin / 1 mM EDTA for 3 – 5 minutes at 37°C. Cells were harvested, washed in MEM and further expanded at a density of 50 cells/cm².

**T cells**

Isolation of CD8^+^CD28^+^ and CD8^+^CD28^-^ T cells from peripheral blood of apparently healthy young (<35y, n=6, mean age 29, range 26-35) and elderly (>65y, n=10, mean age 72, range 66-87) donors was performed by preparing peripheral blood mononuclear cells (PBMCs) by Ficoll-Paque PLUS (Amershan Biosciences) density gradient centrifugation as approved by the local ethics committee. CD8^+^ T cells were negatively selected from the obtained PBMC fraction by applying the magnetic separation protocol CD8^+^ T cell isolation kit II (depleting CD4, CD14, CD16, CD19, CD36, CD56, CD123, TCRγ/δ and CD235a, Miltenyi Biotec) according to the manufacturer’s instructions. Subsequently, purified CD8^+^ T cells were stained with an Allophycocyanin (APC)-conjugated αCD28 monoclonal antibody (mAb) and split into CD8^+^CD28^+^ and CD8^+^CD28^-^ T cell populations using αAPC MicroBeads (Miltenyi Biotec) by passing the cell suspension through a positive selection column (LS; Miltenyi Biotec) mounted in a magnetic field. The CD8^+^CD28^-^ T cell fraction was then reincubated with αAPC MicroBeads and run over a fresh LS-column to increase purity. For phenotypic analysis, purified T cell fractions were labelled with a combination of mAbs (αTCRαβ-FITC, αCD16-PE, αCD4-PerCP, αCD8-PE-Cy7, αCD28-APC and αCD3-APC-Cy7; all BD Biosciences - Pharmingen) and analyzed on a FACSCanto II (BD Biosciences) revealing that the described isolation protocol yields population homogeneities of >95%.

**Foreskin**

Foreskins from donors of different age were frozen in liquid nitrogen and grinded in a mortar. Each sample was lysed in 2 mL Trizol (Invitrogen), and RNA was prepared as described below.

**Microarray Analysis of miRNA Expression**

**RNA Extraction**

For two channel microarray analysis of miRNA expression 0.5 to 1 µg of high quality total RNA were used for labeling. Briefly, total RNA was extracted from cells using Trizol reagent (Invitrogen, CA). 10^6^ - 5*10^6^ cells were pelleted at 900*xg* for 10 minutes and homogenized in 1 mL Trizol (Invitrogen) by vigorous mixing on a vortex (15 sec) followed by incubation at RT for 5 minutes. 200 µL Chloroform were added per 1 mL Trizol, and mixed on a vortex for 15 seconds followed by 3-minute incubation at room temperature. Samples were then centrifuged at 12000*xg* and 4°C for 15 minutes. The upper (aqueous) phase was transferred to a new RNase-free tube and precipitated with 0.5 mL isopropanol (100%, room temperature) per 1 mL initial volume of Trizol. Samples were incubated 10 minutes at room temperature, followed by centrifugation at 12000*xg* for 10 minutes (4°C). The supernatant was discarded and the RNA pellet was washed with 1 mL 75% EtOH (room temperature), and subsequently centrifuged at 7500*xg* for 5 minutes. Ethanol was discarded and the RNA pellet was dried for 10 minutes at room temperature in a fume hood. For resuspension of RNA pellets 20 µL RNase-free water were used. To increase solubility resuspended RNA was incubated at 60°C for 10 minutes. RNA concentration was quantitated using NanoDrop (ThermoScientific, Wilmington, USA) and quality was assessed by testing ribosomal RNA integrity (RNA 6000 Nano Kit, Agilent, Germany). RNA with integrity numbers (RIN) equal or greater than 7 were subsequently stored at -80°C.

**In-house LNA MiRNA Chips (MRC)**

MiRNA Microarrays were spotted on epoxy-coated Nexterion glass slides (Schott AG, Germany) using the MicroGrid II (Zinsser Analytic, Germany) Microarrayer and miRBase version 8.0 or 9.2 locked nucleic acid probe set (Exiqon Inc., Denmark), respectively. The spotted probe set consisted of 559 human and 170 murine miRNAs as well as of 77 miRPlus (intellectual property of Exiqon Inc, Denmark) sequences, which were spotted in 8 replicates on each array. Spotting was performed according to the supplier´s instruction manual and recommendations provided by Exiqon. Subsequently, in order to assure the quality of spotted miRNA microarrays, scanning of all slides was performed. Weak buffer autofluorescence confirmed the presence of more than 98% of spots.

**RNA Hybridization**

For two channel microarray analysis of miRNA expression 0.5 to 1 µg of high quality total RNA were used for labelling using the miRCURY LNA miRNA Array labelling kit (Exiqon, Inc; unless mentioned, all reagents were purchased from Exiqon). Following calf intestinal phosphatase cleavage for 30 minutes at 37°C, labelling with Cy3 and Cy5 fluorescent dyes was performed in a PCR thermocycler at 16°C for 1 hour followed by incubation for 15 minute at 65°C to stop the reaction. Cy3 and Cy5 labelled samples were pooled and mixed with nuclease free water and 2x hybridization buffer to yield 90 µL of hybridization sample in 1x hybridization buffer. Prior to hybridization samples were denatured at 95°C for 2 minutes and snap cooled on ice. RNA samples were then hybridized at 60°C for 16 hours to in-house spotted LNA miRNA chips using a TECAN HS 400 hybridization station (Tecan, Switzerland). Following hybridzation, washing and drying of LNA miRNA microarrays, arrays were immediately scanned using a GenePix 4000B laser scanner and GenePixPro 4.1 software (Axon Instruments). Cy3 dye was scanned at 532nm and Cy5 at 635nm. Scanning settings were adjusted to 10µM resolution and averaging per 1 line. Photomultiplier settings were adjusted individually for Cy3 and Cy5 according to the results from preview scans.

**Microarray data analysis**

**R/Bioconductor**

Intensity values for each spot were extracted using GenePix 4.1 software and inserted into R. Bioconductor and the “Linear models for microarray data analysis” package (Smyth, 2004) were used for the calculation of M-A values for each spot, where M stands for logarithmic red/green ratio (log_2_[R/G]) and A for average log_2_ intensity (log_2_[R*G]*0.5). The MA dataset of each array was background corrected using normexp algorithm and lowess (local weighted linear regression) normalized. Normalized data compared to raw data was visualized using MA and PrintTip Boxplot diagnostic plots as well as density plots (Smyth & Speed, 2003). Subsequently, in order to calculate fold changes in miRNA expression in replicated or aged samples, the 8 replicate spots of each miRNA per array were correlated and linear models were fitted to the data (Smy*th et a*l., 2005). Contrast matrices were defined individually for each experiment, and moderated hypothesis tests (moderated t-statistic) were performed for the contrasts of interest. The data was then adjusted for multiple testing (p-adj.), by controlling the false discovery rate according to a method of Benjamini and Hochberg. All miRNAs were then ranked in terms of their adjusted p-values and a cut-off of p-adj. ≤ 0.05 was imposed (Hochberg & Benjamini, 1990).

**Clustering and Visualization of Array Data**

In order to cluster and visualize log_2_-transformed ratios (LFC) in a heatmap, the freely available Genesis software (Stu*rn et a*l., 2002) was used. The LFCs of 347 miRNAs (corresponding to the intersection between miRBase v8.0 and v9.2 arrays, plus 77 proprietary miRPlus sequences from Exiqon) were inserted in Genesis and, in a second step, filtered for miRNAs which exhibited log_2_-fold changes greater than the average fold change plus 1.5 fold the standard deviation in at least 4 out of 7 experiments (corresponding to a LFC of +/- 0.4).

For identification and visualization of miRNAs regulated in both replicative and organismal aging, the web-based BioVenn application was used (Huls*en et a*l., 2008).

**Quantitative miRNA LNA-PCR and mRNA PCR**

Isolated total RNA from all samples was brought to a concentration of 10 ng/µL and 2 ng/µL in RNAse-free water and quantitative PCR (qPCR) was performed on selected miRNAs and on the internal housekeeping gene glyceraldehyde-3-phosphate dehydrogenase (GAPDH) as well as p21/CDKN1A using a RotorGene 6000 Real-Time Cycler (Qiagen, Germany).

For each miRNA, 9 ng total RNA preparation (4.5 µL) were reverse transcribed in a LNA first strand cDNA synthesis reaction using the miRCURY LNA miRNA PCR System (Exiqon, Inc.) in combination with validated LNA PCR primers (Exiqon, Inc.). Subsequent to 1:10 dilution of the obtained cDNA, 4 µL were used for quadruplicate miRNA PCR reaction according to recommendations of the manufacturer. For GAPDH housekeeping gene as well as p21/CDKN1A, 70 ng total RNA were reverse transcribed using MMLV-reverse transcriptase (Finnzymes, Finnland) and random hexamer primers. Following 1:3 dilution in nuclease-free water, 1 µL cDNA was qPCR amplified in quadruplicate using SensiMix Plus SYBR Green (Quantace, Germany) and specific primers (250 pM final concentration). To ensure appropriate size of the amplification products, melting points were determined after each amplification. For copy number determination of mRNAs, internal standards were included in each run. These were derived from PCR amplification from human cDNA and subsequent PCR product purification (Promega) and quantitation. Six standards in the range from 10^8^ to 10^3^ copies/µL were included in each run. The resulting standard curve was calculated in Rotor-Gene 6000 Series Software 1.7 and used for quantification of copy numbers of mRNA targets.

Fehrer C, Brunauer R, Laschober G, Unterluggauer H, Reitinger S, Kloss F, Gully C, Gassner R, Lepperdinger G (2007) Reduced oxygen tension attenuates differentiation capacity of human mesenchymal stem cells and prolongs their lifespan. *Aging Cell* **6,** 745-757.

Hochberg Y, Benjamini Y (1990) More powerful procedures for multiple significance testing. *Stat Med* **9,** 811-818.

Hulsen T, de Vlieg J, Alkema W (2008) BioVenn - a web application for the comparison and visualization of biological lists using area-proportional Venn diagrams. *BMC Genomics* **9,** 488.

Jaffe EA, Nachman RL, Becker CG, Minick CR (1973) Culture of human endothelial cells derived from umbilical veins. Identification by morphologic and immunologic criteria. *J Clin Invest* **52,** 2745-2756.

Smyth GK (2004) Linear models and empirical bayes methods for assessing differential expression in microarray experiments. *Stat Appl Genet Mol Biol* **3,** Article3.

Smyth GK, Michaud J, Scott HS (2005) Use of within-array replicate spots for assessing differential expression in microarray experiments. *Bioinformatics* **21,** 2067-2075.

Smyth GK, Speed T (2003) Normalization of cDNA microarray data. *Methods* **31,** 265-273.

Sturn A, Quackenbush J, Trajanoski Z (2002) Genesis: cluster analysis of microarray data. *Bioinformatics* **18,** 207-208.

Wieser M, Stadler G, Jennings P, Streubel B, Pfaller W, Ambros P, Riedl C, Katinger H, Grillari J, Grillari-Voglauer R (2008) hTERT alone immortalizes epithelial cells of renal proximal tubules without changing their functional characteristics. *Am J Physiol Renal Physiol* **295,** F1365-1375.
